# Altered Proteolysis in Fibroblasts of Alzheimer Patients with Predictive Implications for Subjects at Risk of Disease

**DOI:** 10.1155/2014/520152

**Published:** 2014-05-18

**Authors:** Alessandra Mocali, Nunzia Della Malva, Claudia Abete, Vito Antonio Mitidieri Costanza, Antonio Bavazzano, Vieri Boddi, Luis Sanchez, Sandra Dessì, Alessandra Pani, Francesco Paoletti

**Affiliations:** ^1^Section of Experimental Pathology and Oncology, Department of Biomedical Experimental and Clinical Sciences, University of Florence, 50134 Florence, Italy; ^2^Department of Internal Medicine, University of Cagliari, 09042 Monserrato, Italy; ^3^Geriatric Unit of ASL 4, Prato Hospital, 59100 Prato, Italy; ^4^Department of Public Health, University of Florence, 50134 Florence, Italy; ^5^1st Unit of General Surgery and Transplantation, Careggi Hospital, 50134 Florence, Italy; ^6^Department of Biomedical Sciences, University of Cagliari, 09042 Monserrato, Italy

## Abstract

There is great interest in developing reliable biomarkers to support antemortem diagnosis of late-onset Alzheimer's disease (AD). Early prediction and diagnosis of AD might be improved by the detection of a proteolytic dysfunction in extracts from cultured AD fibroblasts, producing altered isoelectrophoretic forms of the enzyme transketolase (TK-alkaline bands). The TK profile and apolipoprotein E (APOE) genotype were examined in fibroblasts from 36 clinically diagnosed probable late-onset sporadic AD patients and 38 of their asymptomatic relatives, 29 elderly healthy individuals, 12 neurological non-AD patients, and 5 early-onset AD patients. TK alterations occurred in (i) several probable AD patients regardless of age-of-onset and severity of disease; (ii) all early-onset AD patients and APOE **ε**4/4 carriers; and (iii) nearly half of asymptomatic AD relatives. Normal subjects and non-AD patients were all negative. Notably, culture conditions promoting TK alterations were also effective in increasing active BACE1 levels. Overall, the TK assay might represent a low-cost laboratory tool useful for supporting AD differential diagnosis and identifying asymptomatic subjects who are at greater risk of AD and who should enter a follow-up study. Moreover, the cultured fibroblasts were confirmed as a useful *in vitro* model for further studies on the pathogenetic process of AD.

## 1. Introduction

A definite diagnosis of Alzheimer's disease (AD) can be accomplished only post-mortem by histopathology of the autopsied brain so as to reveal amyloid beta protein (A**β**) in neuritic plaques and intraneuronal neurofibrillary tangles [1, 2]. In past years several alterations in peripheral cells and biological fluids of AD patients have been proposed as potential antemortem AD biomarkers [3–6]. However, none have met the criteria established for an ideal biomarker [7, 8], capable of assessing whether a mild cognitive impairment (MCI) might reveal early stages of disease [9] or should rather be linked to normal aging. In fact, advances in our knowledge of AD have shown that symptoms usually develop after a long preclinical pathogenetic process, making early detection of AD in asymptomatic subjects of great interest. Indeed, the assessment of presymptomatic subject cohorts regardless of genetic predisposition [10, 11], including relatives of AD patients, could be highly useful in determining the effectiveness of pharmacological intervention in slowing AD onset and/or cognitive decline in AD patients.

Clinical criteria for AD diagnosis have recently been revised [12] and the terms* possible* and* probable* AD have been redefined by including some biomarkers for the pathophysiological process in support of AD diagnosis. However, their use has been limited to research settings due to the fact that these procedures are not easily available to all patients. The combination of multiple parameters [5, 13] obtained through neuropsychological testing and conventional and functional imaging [14, 15], together with the relatively low accuracy attainable in discriminating AD from other dementia [15], requires elaborate, serial, and costly procedures, available only in the best academic centers and for a limited number of patients. Therefore, there is a great need of reliable and low-cost peripheral markers to identify persons with incipient AD and complement clinical AD outcomes in a large number of subjects. The National Institute on Aging and Alzheimer's Association international workgroup recently reviewed the state-of-the-art in this field to develop recommendations to determine which factors best predict the risk of progression from “normal” cognition to mild cognitive impairment (MCI) and AD dementia [16]. A combination of CSF T-tau and A**β**42/P-tau ratio has shown to be useful for defining MCI patients at high risk of developing AD, yielding a positive predictive value of 81% [17]. The various constituents of blood, including plasma, platelets, and cellular fractions [18], are now being systematically explored as a pool of putative peripheral biomarkers for AD for the ease of sampling and repeatability of measures [15, 19]. In this context, disease-specific autoantibody profiles [20], increased glutaminyl cyclase expression [21], and angiopoietin-1 levels [22]have also been described in human sera. On the whole, all these findings need verification in further studies [23, 24]; nevertheless they lend further support to the hypothesis that AD patients suffer from a systemic metabolic dysfunction that, in addition to the brain, also affects peripheral tissues.

Fibroblasts have also often been employed as an* in vitro *model for neurological diseases [26] and, particularly, for AD [4, 25, 27]. For instance, AD fibroblasts, unlike their normal counterpart, display a variety of alterations related to GM1 ganglioside catabolism [28], the function of K+ channel [29] and Ca++ homeostasis [30], Erk1/2 levels in response to bradykinin [6], cholesterol ester cycle [31], and, eventually, dysregulated proteolysis [32–36]. Recently, a fibroblast biomarker profile was proposed to identify accurately AD patients for therapeutic intervention [37]. A very attractive approach could be that of using patient fibroblasts reprogrammed to neurons and exhibiting AD phenotype [38] as a model.

Herein, we report that early prediction and diagnosis of AD might be improved by measuring typical proteolytic alterations that we formerly found in cultured AD fibroblasts [25]. These alterations involve transketolase (TK), a thiamine-dependent enzyme that undergoes limited degradation by the enhanced activities of cysteine proteinases released upon cell extraction [32, 33], to yield isoforms with unusually high alkaline pI (TK-alkaline bands, TK-ab).

In this paper, for the first time subjects from 36 families of probable late-onset AD patients were analyzed for the frequency and intensity of the TK-ab signal, and for the APOE genetic asset. Then, statistic analyses were carried out to correlate TK-ab positivity and the presence of APOE *ɛ*4 allele. Remarkably, asymptomatic first-degree relatives were recruited and identified as subjects at greater risk of developing AD [39], independently of their APOE genetic asset.

These subjects are often overlooked in most studies focused on detection of disease biomarkers without considering that AD relatives would probably be the most cooperative and interested individuals for investigation with noninvasive analyses.

Healthy subjects, patients with other non-AD neuropathologies, and early-onset AD patients were also analyzed. Our results reveal TK-ab as a steady indicator of cultured AD fibroblasts, that is, associated significantly with both late- and early-onset forms of AD regardless of age-of-onset or stage of disease. It could also be of prognostic value, helpful in assessing AD risk in single individuals and applicable to asymptomatic relatives of AD patients and APOE-*ε*4 carriers [40].

BACE1 is the key rate-limiting enzyme for the production of the A**β** peptide and its activity has been found to increase under starvation* in vitro* [41] and after energy inhibition in APP transgenic mice [42]. The amount of active BACE1 increased significantly in extracts of cultured fibroblasts positive to the Tk-ab test, supporting the usefulness of cultured fibroblasts as an excellent* in vitro *model for the study of the pathogenetic process of AD.

## 2. Patients and Methods

### 2.1. Subjects

Individuals who entered the study have been enrolled in the Geriatric Unit of Prato Hospital (Italy) and included (a) elderly healthy subjects (controls, *n* = 29, mean age ± SD = 66.8 ± 11.1, age range: 50–86); (b) patients with a clinical diagnosis of probable late-onset or sporadic AD (*n* = 36, mean age ± SD = 70.8 ± 7.3, age range: 61–86); (c) asymptomatic first-degree relatives of AD patients (*n* = 38, mean age ± SD = 46.7 ± 10.9, age range: 32–68); and (d) neurological non-AD patients (*n* = 12, mean age ± SD = 67.2 ± 10.5, age range: 50–84) including 4 patients with vascular dementia, 3 patients with frontotemporal dementia, 2 patients with Parkinson's disease, 1 patient with severe neurosis, 1 patient with intermittent ataxia, and 1 patient with olivopontocerebellar ataxia. Clinical diagnosis of probable AD was made according to the criteria established by the Diagnostic and Statistical Manual of Mental Disorders (4th edition, DSM IV) [43], the National Institute of Neurological and Communicative Disorders and Stroke, and the Alzheimer's Disease and Related Disorders Association (NINCDSADRDA) [44] and reevaluated according to the NIA-Alzheimer's Association workgroups on diagnostic guidelines for AD [12]. Medical examinations by neurologists with the aid of neuropsychological and laboratory tests and sometimes computed tomography or magnetic resonance of the brain were performed. The mini-mental state examination (MMSE) [45] and global deterioration scale (GDS) [46] were used as the staging systems. Informed written consent to use blood and dermal cells for analysis was obtained from the subjects or, when necessary, from their legal guardians under local institutional review board supervision and approval. Further, we analyzed fibroblasts (kindly provided by S. Sorbi, Department of Neurology, University of Florence) from early-onset AD patients (*n* = 5, mean age ± SD = 48.0 ± 6.9, age range: 38–55) carrying mutations of APP (Val717Ile, 1 case), presenilin 1 (PS1) (Met146Leu, 2 cases), and presenilin 2 (PS2) (Met239Val, 2 cases).

### 2.2. Skin Biopsies, Fibroblast Cultures, and Immunodetection of TK-Isoforms

**Figure 1:**
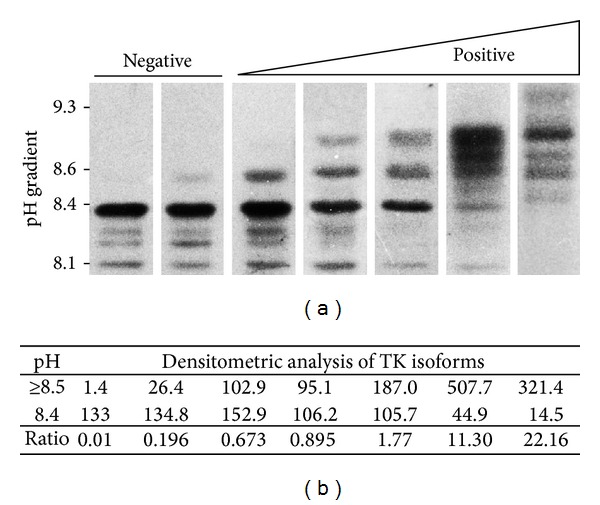
The most representative typical and altered isoelectrophoretic (IEF) profiles of TK from cultured human fibroblasts. (a) Aliquots (30–40 *μ*L; 1 mg/mL) of fibroblast extracts were prepared and separated by IEF within a pH range of 3–10 and then blots were probed with anti-human TK antibody (see Section 2 and [25]). Negative samples presented no distinct band or just a faint signal over pH 8.4, while positive samples exhibited one to three heavily stained TK-ab isoforms migrating toward the alkaline region of gel (pH range of 8.5–9.4). (b) Densitometric analysis of immunostained TK was carried out with the aid of the ImageJ software and values of intensity were expressed as arbitrary units; the ratio between values of TK-ab (pH ≥ 8.5) and those of the normal TK band (pH = 8.4) for each sample is reported. The border between negative and positive samples was arbitrarily set at a ratio of 0.4. TK, transketolase.

Skin biopsies were obtained from the forearm of subjects by a 2 mm punch, under local anesthesia with 2% xylocaine; healing was usually complete within a week. Tissue fragments were placed in culture and fibroblasts were then propagated under culture conditions favoring TK-ab expression. Briefly, cells were seeded using a medium at pH 7.8 [47] and maintained for 2 weeks without medium changes [32]. Then fibroblasts were harvested and lysed by sonication and cell extracts were separated by isoelectric focusing (IEF) as previously reported [33]. Separated proteins were transferred to a nitrocellulose membrane and then probed with rabbit polyclonal IgG raised against human TK [48]. TK-isoforms were detected by either carbazole staining (Sigma, St. Louis, MO) or ECL procedure (Amersham Biosciences) [25, 33] and quantified by densitometric analysis with the aid of ImageJ software (see Section 3 and legend in Figure 1).

### 2.3. APOE Genotyping

Peripheral blood mononuclear cells were isolated by centrifugation at 1,700 g on Lymphoprep (Eurobio, Les Ulis Cedex B, France). Genomic DNA was then extracted by using the NucleoSpin Tissue kit (Macherey-Nagel Gmbh & Co. KG, Düren, Germany). DNA was amplified by PCR in a DNA thermal cycler (GeneAmp PCR System 2700, Applied Biosystems, NJ) as reported by Hixson and Vernier [49].

### 2.4. BACE1 Determination

Fibroblasts from two healthy aging control subjects (Cont 1 and Cont 2), from six probable AD patients (AD1-6), and from two asymptomatic first-grade AD relatives (Rel 1 and Rel 2) were analyzed. Cells were seeded and maintained in parallel under conditions favoring TK-ab production (see above), called “positive,” and under normal conditions (with normal medium pH and detached at confluence), called “negative.” Harvested fibroblasts were extracted with RIPA buffer (R0278, Sigma, 0.1 mL/1,000,000 cells) and protein concentration was assessed by the bicinchoninic acid protein determination kit (Sigma). For BACE1 determination, aliquots (20 *μ*g) of total cell extracts were separated on 4–12% NuPAGE Bis-Tris Gels with MOPS-SDS running buffer (Novex, Invitrogen, Carlsbad, CA) and blots were probed with anti-BACE1 antibody (NB120-10716, Novus Biologicals, Littleton, CO). After incubation with HRP-conjugated secondary antibodies, specific bands corresponding to BACE1 proteins were detected by the ECL procedure (Amersham, Freiburg, Germany) and quantified by densitometric analysis with the aid of ImageJ software. Anti-GAPDH antibodies (Cell Signaling, Beverly, MA) were used for detection of the housekeeping protein.

### 2.5. Statistics

TK-ab expression and APOE genotype frequencies in different groups of subjects were compared by means of Fisher's exact test. The analysis of variance test (ANOVA) was used to establish whether the degree of AD severity or patient age differed significantly between TK-ab-positive and -negative cases. Significance level was set at *P* = 0.05. Sensitivity, specificity, accuracy, and predictive values (PV) of TK-ab expression were reported together with 95% confidence intervals (CI).

## 3. Results and Discussion

### 3.1. TK Analysis in Cultured Fibroblasts from Different Groups of Subjects

Altered TK-isoforms (TK-ab) have frequently been observed in extracts of cultured fibroblasts from AD patients and proposed as potential indicators of disease (see Section 1 and [25, 32, 34]). The conditions for optimal expression and detection of TK-ab following IEF separation have been reported elsewhere [32, 47]; however, for the sake of clarity, a panel of the typical and of the most representative altered TK profiles as well as the densitometric criteria used to evaluate results of the analysis were reported (Figures 1(a) and 1(b), resp.). The protein band migrating at pH 8.4 was the major TK isoform in control samples, together with at least three minor and less basic bands. Instead, TK-ab exhibited a more alkaline pI and migrated within a pH range of 8.5–9.4. The presence of TK-ab was usually assessed by visual inspection; however, to decide on uncertain cases a densitometric analysis of immunostained bands was employed. Intensity values of TK-ab (pH ≥ 8.5) were calculated and divided by those of the normal TK band (pH = 8.4); the border between negative and positive samples was arbitrarily set at a ratio of 0.4. Of note, a strong positive TK-ab signal (pH ≥ 8.5) as observed for many of the AD patients was usually accompanied by a decrease in size and staining of the major TK band (pH = 8.4).

**Figure 2:**
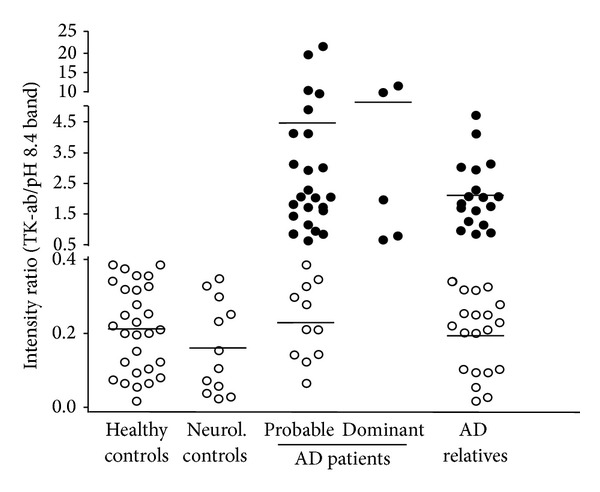
TK-ab determination in cultured dermal fibroblasts. Total protein extracts from 29 healthy control subjects, 12 neurological non-AD patients (neurol controls), 36 probable AD patients, 5 early-onset (dominant AD) patients, and 38 first-degree relatives of AD patients (AD relatives) were analysed for TK-ab expression as described in Figure 1. TK-ab-negative and -positive cases are represented by open and closed circles, respectively.

TK profiles were analyzed in fibroblasts derived from 5 distinct groups of individuals and results are reported in Figure 2 (for details see legend and Section 2). The number of TK-ab-positive cases was markedly high among probable AD patients while both healthy controls and neurological non-AD patients (neurol. controls) were all TK-ab negative. Based on these findings, TK-ab could be validated as a potential peripheral AD signature, showing a sensitivity = 69.4% (CI = 51.9–83.6), a specificity = 100% (CI = 91.4–100), and an accuracy = 85.7% (CI = 75.9–92.6). The negative or positive predictive value (PV) was 78.8% (CI = 65.3–88.9) or 100% (CI = 86.3–100), respectively. TK-ab-positive and -negative cases did not differ significantly with regard to age-at-onset (analysis of variance: *P* = 0.76) or disease severity as determined by GDS [46] and MMSE [45] clinical tests (*P* = 0.20 and *P* = 0.26, resp.). Notably, early-onset AD patients (dominant AD) carrying APP, PS1, and PS2 mutations were all TK-ab positive. Nearly half (47.4%) of asymptomatic first-degree AD relatives (mean age <50) turned out to be TK-ab-positive. There was a striking statistically significant difference in TK-ab expression in either possible AD patients or AD relatives (*P* < 0.0005 for both subsets)* versus *healthy controls. This result indicated a clear inclination of AD relatives toward the distribution pattern of AD patients and supported the view that individuals who have had an AD patient in the family run a major risk of developing AD [39]. In particular, we found the percentage of TK-ab positivity in our group of relatives at a level very near to that expected for the dominantly inherited AD [50]. In this regard, our cohort of patients had only a clinical diagnosis of probable late-onset or sporadic AD, but we are inclined to exclude the presence of inherited dominant mutations in relatives, given the high level of age-at-onset of the disease (age range 65–82) in patients. The TK-ab positive relatives could conceivably have a predisposing genetic and/or environmental asset; this might require further analyses.

### 3.2. Cross-Evaluation of TK-ab Expression and APOE Genotype

Although our sample size is small, we have attempted to establish whether the APOE-*ε*4 genotype, a well-known risk factor for AD, was potentially related to TK-ab expression. The answer to this question is negative; no statistically significant correlation was found between TK-ab expression and APOE genotype in either AD relatives or AD patients (*P* = 0.746 and *P* = 0.159, resp.) which would infer that these parameters address distinct pathogenetic mechanisms.

### 3.3. Cross-Evaluation of TK-ab Expression and APOE Genotype in AD Relatives

The cross-evaluation of TK-ab positivity and APOE genotype was carried out within AD relatives to assess whether it might improve AD prediction within these relatively young and asymptomatic subjects. Results showed that the two homozygous *ε*4/4 carriers were both TK-ab-positive. Among heterozygous *ε*3/4 carriers there were 5 out of 10 TK-ab-positive cases; finally, TK-ab-positive cases were highly represented among *ε*3/3 (11 out of 21) and also one *ε*2/3 carrier was positive. An increased number of positive cases within *ε*3/3 relatives of AD patients could have been expected [39], and TK-ab positivity did not improve the prediction of AD risk based on *ε*4 allele frequency [40] at the population level, but it might be crucial to identifying those subjects who, although asymptomatic, express TK-ab at early ages and are, conceivably, at higher risk of progressing to AD.

### 3.4. Application of the Combined APOE/TK-ab Tests to Families of Probable AD Patients

**Figure 3:**
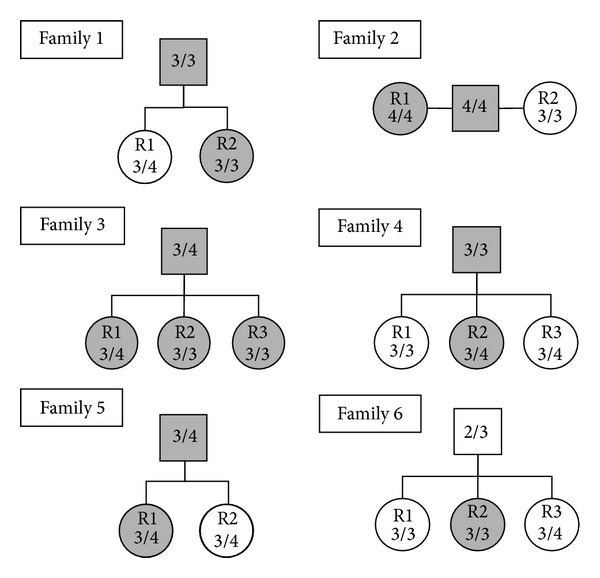
Combined APOE/TK-ab tests in 6 families of probable AD patients. Combined results of the clinical diagnosis and genetic and biochemical tests in members of 6 representative families, each with a probable AD patient (square symbol) and 2 to 3 relatives (R). TK-ab positivity corresponded to closed symbols. APOE genotypes are indicated inside symbols.

The combined results of the clinical diagnosis and genetic and biochemical tests in members of 6 out of the 36 families examined are shown in Figure 3, where each family is represented by a probable AD patient and 2 to 3 relatives (R).

*Family 1*. (AD, female, 70 years, onset at 67; R1, female, 43 years; R2, male, 41 years) R2 son, for its positivity to TK-ab test, could be considered at higher risk of AD, despite the fact he had a more protective APOE genetic asset than R1.

*Family 2*. (AD, female, 74 years, onset at 72; R1, female, 71 years; R2, female, 70 years) represented by a possible AD patient and two sisters: it was confirmed that *ε*4/4 subjects were always positive to the TK-ab test whether they were clinically diagnosed as AD patients or still unrecognized as in the case of R1 who, due to *ε*4/4 homozygosity, will conceivably develop AD in subsequent years.

*Family 3*.  (AD, female, 86 years, onset at 78; R1, male, 58 years; R2, male, 54 years; R3, female, 50 years). All three sons were TK-ab positive, indicating that the presence of the marker was a constant trait in this family; evidently, the whole genetic asset conferred the same risk related to altered proteolysis to the three Rs irrespective of *ε*4 allele frequency that might play a role in determining the age of onset.

*Family 4*. (AD, female, 81 years, onset at 79; R1, female, 55 years; R2, male, 51 years; R3, male, 39 years). The positivity to TK-ab test addressed R2 as the subject with highest probability of developing late-onset AD, that in principle should have been equally shared by the two sons (R2 and R3), both carrying the APOE *ε*3/4 genetic asset.

*Family 5*. (AD, male, 81 years, onset at 77; R1, female, 49 years; R2, male, 53 years): as in family 4, our results indicate R2 as the subject with the highest risk of developing late-onset AD.

*Family 6*. (AD, female, 67 years, onset at 66; R1, female, 40 years; R2, female, 36 years; R3, male, 34 years). The AD patient, for both the APOE genetic asset and the absence of Tk-ab trait, should be clinically reevaluated for other neuropathologies; R2 seems to be the only subject with the highest probability of developing late-onset AD irrespective of the absence of *ε*4 allele.

### 3.5. BACE1 Determination and Activity following Culture Conditions Producing TK-ab Forms

**Figure 4:**
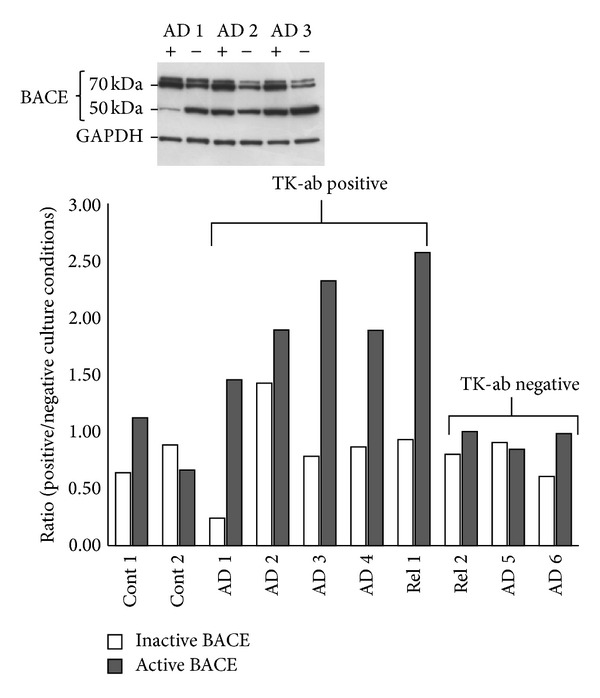
BACE1 activation by fibroblast culture conditions. Fibroblasts from two healthy aging control subjects (Cont 1 and Cont 2), from 6 probable AD patients (AD1-6), two of whom resulted negative to the TK-ab test (AD5 and AD6), and from two AD relatives (Rel 1 and Rel 2) were cultured under conditions favoring TK-ab production (positive) and under normal conditions (negative). Three immunoreactive bands of BACE1 protein were detected, corresponding to the precursor protein (inactive BACE1) (~50 KDa) and to two glycosylated active enzyme forms (active BACE1, within the range of 70–80 KDa), as shown in the insert. BACE1 proteins were quantified by densitometric analysis using GAPDH as the housekeeping; then the ratio between protein amounts in positive and negative culture conditions was calculated and reported for each subject.

BACE1 is the key rate-limiting enzyme for the production of the A**β** peptide and its activity has been found to increase under starvation* in vitro* [41] and after energy inhibition in APP transgenic mice [42]. In order to assess whether our fibroblast samples might show other well-known AD metabolic alterations, that could be related to TK-ab or not, BACE1 activity was determined in extracts of cultured fibroblasts which were maintained in parallel under culture conditions “positive” and “negative” for TK-ab production (see Section 2). The results of a preliminary experiment are shown in Figure 4: two healthy aging control subjects (Cont 1 and Cont 2), two AD relatives (Rel 1 and Rel 2), and 6 probable AD patients (AD1-6) were analyzed for BACE1 electrophoretic pattern. Three immunoreactive bands of BACE1 protein were detected (see insert): (i) one indicated the precursor protein (immature BACE1, ~50 KDa) and (ii) two forms corresponded to the glycosylated active enzyme (mature BACE1, within the range of 70–80 KDa) [51, 52]. We found that the amount of the active enzyme increased significantly under positive culture conditions in comparison to negative ones (ratio > 1), in fibroblast extracts which contemporarily yielded a positive Tk-ab signal.

## 4. Conclusions

We propose that the TK-ab test may be used as a peripheral indicator of disease in fibroblasts from AD patients in this study. The sensitivity of this test (as opposed to specificity, accuracy, and predictive values) was not extraordinarily high but it should be remembered that these individuals were diagnosed as probable AD patients only by standard clinical criteria. There are, however, some aspects concerning the significance and applications of TK analysis that deserve further comments.

First, the TK-ab signature seems to be independent of age-of-onset, severity, and form of AD. Such a steady expression from the early to the late clinical stages of disease is a distinctive feature of TK-ab as compared to other peripheral AD indicators. In fact, even A**β** peptides, the most sensitive and direct hallmark of AD, increase in plasma of rare dominant AD patients [53] but not in most common late-onset AD patients [54] who actually show a decline in A**β**-42 levels [9, 55].

Secondly, TK-ab could be detected even in the absence of clinical signs such as in (i) approximately half of the subjects younger than 50 who, irrespective of *ε*4 frequency, have had an AD patient in the family and run a major risk of developing AD later in life [39] and (ii) in asymptomatic *ε*4/4 carriers who have a high probability of developing AD with age [40]. The identification of the TK-ab signature in combination with genetic profile in relatives of AD patients—but it might also be worth for subjects with MCI or less clearly determined cognitive deficits—could provide early recognition of at-risk subjects and allow targeted intervention to delay neurodegeneration. We cannot say that all asymptomatic individuals with a single APOE *ε*4 gene dose and positive for TK-ab will necessarily develop the disease at advanced age but, at least, it could be possible to identify those subjects who should enter a follow-up study.

Thirdly, our results suggest that mechanisms underlying TK-ab production and BACE1 activation might be related. In addition, the fact that the amount of active BACE1 increased significantly in extracts of cultured Tk-ab-positive fibroblasts reinforces the specificity of our test.

On the whole, this study supports the usefulness of cultured fibroblasts as an excellent* in vitro *model for the study of the pathogenetic process of AD and for preliminary tests of toxicity and efficacy of agents capable of reestablishing the control of intracellular proteolysis, including BACE1 activation.
